# Unexpected Diversity and Photoperiod Dependence of the Zebrafish Melanopsin System

**DOI:** 10.1371/journal.pone.0025111

**Published:** 2011-09-22

**Authors:** Vanessa Matos-Cruz, Joseph Blasic, Benjamin Nickle, Phyllis R. Robinson, Samer Hattar, Marnie E. Halpern

**Affiliations:** 1 Department of Embryology, Carnegie Institution for Science, Baltimore, Maryland, United States of America; 2 Department of Biology, Johns Hopkins University, Baltimore, Maryland, United States of America; 3 Department of Biological Sciences, University of Maryland, Baltimore County, Baltimore, Maryland, United States of America; Yale School of Medicine, United States of America

## Abstract

Animals have evolved specialized photoreceptors in the retina and in extraocular tissues that allow them to measure light changes in their environment. In mammals, the retina is the only structure that detects light and relays this information to the brain. The classical photoreceptors, rods and cones, are responsible for vision through activation of rhodopsin and cone opsins. Melanopsin, another photopigment first discovered in Xenopus melanophores (Opn4x), is expressed in a small subset of retinal ganglion cells (RGCs) in the mammalian retina, where it mediates non-image forming functions such as circadian photoentrainment and sleep. While mammals have a single melanopsin gene (*opn4*), zebrafish show remarkable diversity with two *opn4x*-related and three *opn4*-related genes expressed in distinct patterns in multiple neuronal cell types of the developing retina, including bipolar interneurons. The intronless *opn4.1* gene is transcribed in photoreceptors as well as in horizontal cells and produces functional photopigment. Four genes are also expressed in the zebrafish embryonic brain, but not in the photoreceptive pineal gland. We discovered that photoperiod length influences expression of two of the *opn4*-related genes in retinal layers involved in signaling light information to RGCs. Moreover, both genes are expressed in a robust diurnal rhythm but with different phases in relation to the light-dark cycle. The results suggest that melanopsin has an expanded role in modulating the retinal circuitry of fish.

## Introduction

Melanopsin, the long sought after photopigment involved in circadian regulation, was first shown to mediate light dependent dispersal of pigment granules in Xenopus melanophores [1] and later found to be produced in the ganglion cell layer of the mammalian retina [2], [3]. Approximately 2–4% of retinal ganglion cells (RGCs) in the mouse retina express melanopsin, where they serve as a specialized class of photoreceptive cells that directly transmit light information to the brain [3]. Much has been learned about the diversity and functions of these intrinsically photosensitive RGCs (ipRGCs) in mammals in regulating circadian activity, the pupillary light response and sleep, as well as contributing to light detection for vision [3], [4], [5], [6], [7]. However, less is known about melanopsin proteins and the roles of melanopsin-expressing cells in non-mammalian vertebrates, especially in aquatic species.

In contrast to mammals that have a single *melanopsin* or *Opsin 4* (*Opn4*) gene, the genomes of birds, amphibia and fish contain genes belonging to two groups that encode either Opn4x-related or Opn4-related proteins on the basis of their greater similarity with either the Xenopus or mammalian protein, respectively [8]. Genomic analysis suggests that, during evolution, mammals lost the *opn4x* gene through a chromosomal rearrangement [8].

In non-mammalian vertebrates, *opn4*-related genes are not only expressed in a small subset of retinal ganglion cells, but also in interneurons of the inner nuclear layer of the retina [1], [9], [10], [11]. In teleost fish, such as the Atlantic cod, cichlid and roach melanopsin transcripts are detected in horizontal cells [10], [12], [13]. *Opn4-related genes are* also expressed in some amacrine cells in the developing and adult retina of the Atlantic cod and chicken [10], [11].

While the retina is the only light-detecting organ in mammals, other vertebrate species have evolved specialized extraocular photoreceptors that allow them to detect changes in irradiance [14]. In some birds and reptiles, for example, both the pineal gland and deep-brain photoreceptors are thought to regulate circadian entrainment and seasonal responses to changes in photoperiod length [14], [15]. A number of photopigments have been localized to extraocular photoreceptors, including neuropsin [16], vertebrate-ancient Opsin (Val-Opsin) [17], parapinopsin [18], pinopsin [19], exo-rhodopsin [20] and melanopsin [1], [9], [10]. In the case of melanopsin, expression is found in the photosensitive pineal organ of chickens, where mRNA levels oscillate in a circadian manner [9]. In various species, melanopsin is also produced in the biological clock, the suprachiasmatic nucleus (SCN), and in other regions of the brain including the habenular nuclei, the thalamus, hypothalamus and the lateral septal organ, the presumed deep-brain photoreceptor of birds [1], [9], [10]. The functional relevance of these diverse sites of melanopsin photoreceptors in the brain is not well understood.

We set out to characterize the melanopsin system in the zebrafish because of its advantages as a vertebrate genetic model. The anatomy, circuitry and biochemistry of the retina are highly conserved across vertebrates, including the zebrafish [21]. By 5 days post-fertilization (dpf), the larval retina has differentiated and is functional, displaying responses evoked by visual stimuli [21], [22], [23]. In previous studies, cDNA clones corresponding to two mammalian-like genes, *opn4a* and *opn4.1* (originally named *opn4m1* and *opnm2*), were identified [8], [24]. Expression analyses by RT-PCR had indicated that *opn4a* is expressed in the adult eye and brain but not in skin and muscle cells [8], [24]. Transcripts for the *opn4.1* gene were also detected by in situ hybridization in horizontal cells in the adult retina [24]. However, there were no reports of any zebrafish *opn4*-related gene expressed in RGCs, as in mammals and other vertebrate species.

Here we describe the five *melanopsin* genes that are present in the zebrafish genome and their diverse expression in multiple neuronal cell types of the developing retina, including classical photoreceptors, horizontal, amacrine and bipolar interneurons and RGCs. Unlike birds [9], [11], transcripts were not detected in the presumptive pineal organ of zebrafish larvae, although a few *melanopsin*-expressing cells were found at the base of the pineal stalk. Expression was also observed in discrete regions of the developing forebrain and hindbrain. We unexpectedly discovered that length of the photoperiod influences expression of two *opn4* related genes, revealing robust rhythms in their levels. The results suggest that zebrafish have evolved an adaptive melanopsin system that may not only mediate non-image forming light responses but also modulate visual input.

## Results

### Five zebrafish *melanopsin* genes arose by gene duplication and retrotransposition

**Figure 1:**
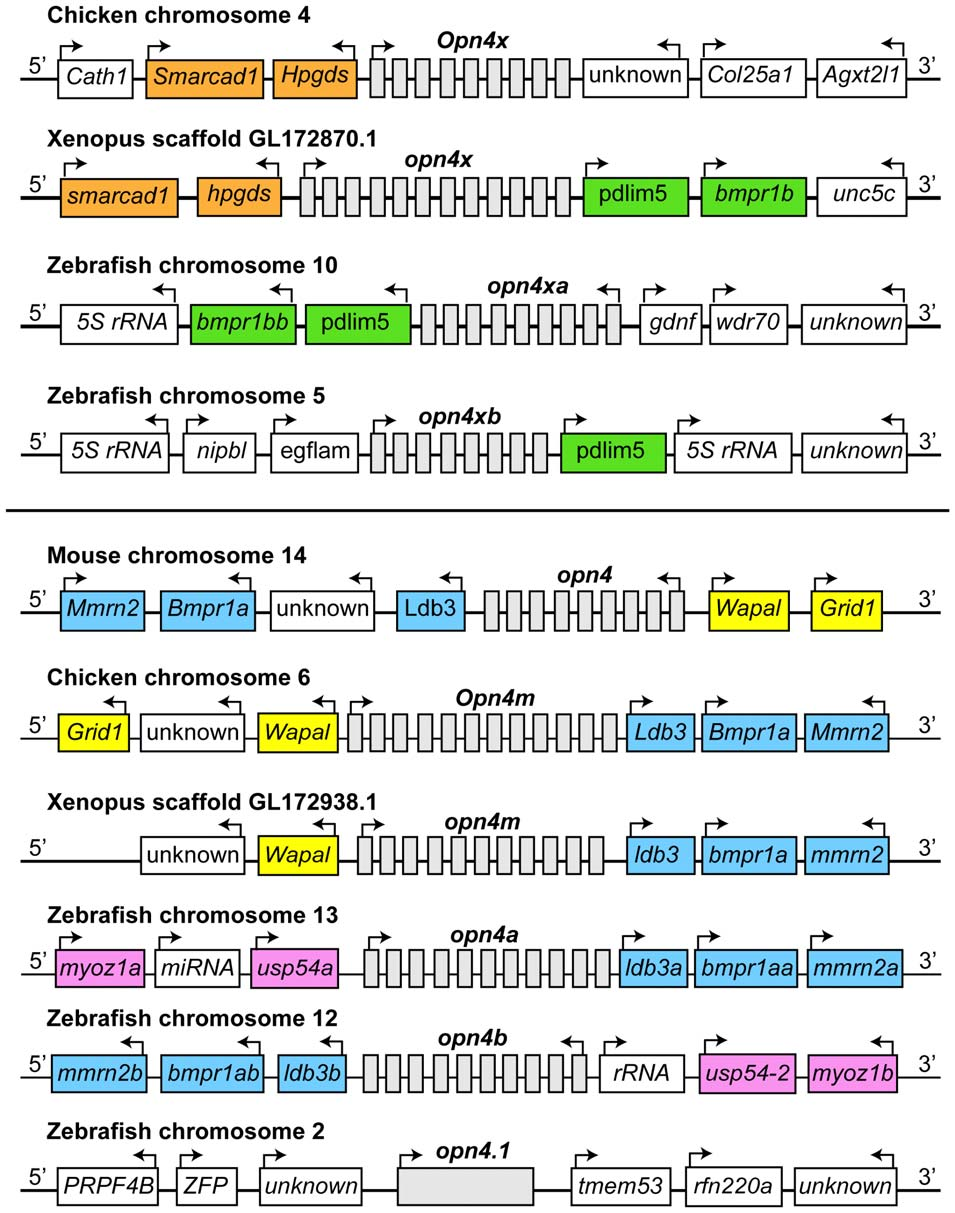
Five zebrafish *opn4*-related genes arose by duplication and retrotransposition. Schematic diagrams of the chromosomal regions surrounding mouse (*Mus musculus*), chicken (*Gallus gallus*), frog (*Xenopus tropicalis*) and zebrafish (*Danio rerio*) *opn4-related* loci. Gray boxes represent *melanopsin* exons and arrows indicate direction of transcription. The *opn4a* and *opn4.1* genes were previously identified [8], [24]. Orange and green boxes represent syntenic genes located upstream and downstream of the chicken *opn4* locus. Blue and yellow boxes represent syntenic genes located upstream or downstream of the mouse *Opn4* locus. Pink boxes represent conserved genes upstream of duplicated zebrafish *opn4m* loci. The single exon *opn4.1* locus shows no synteny with other *opn4* chromosomal regions and likely arose by retrotransposition.

**Figure 2:**
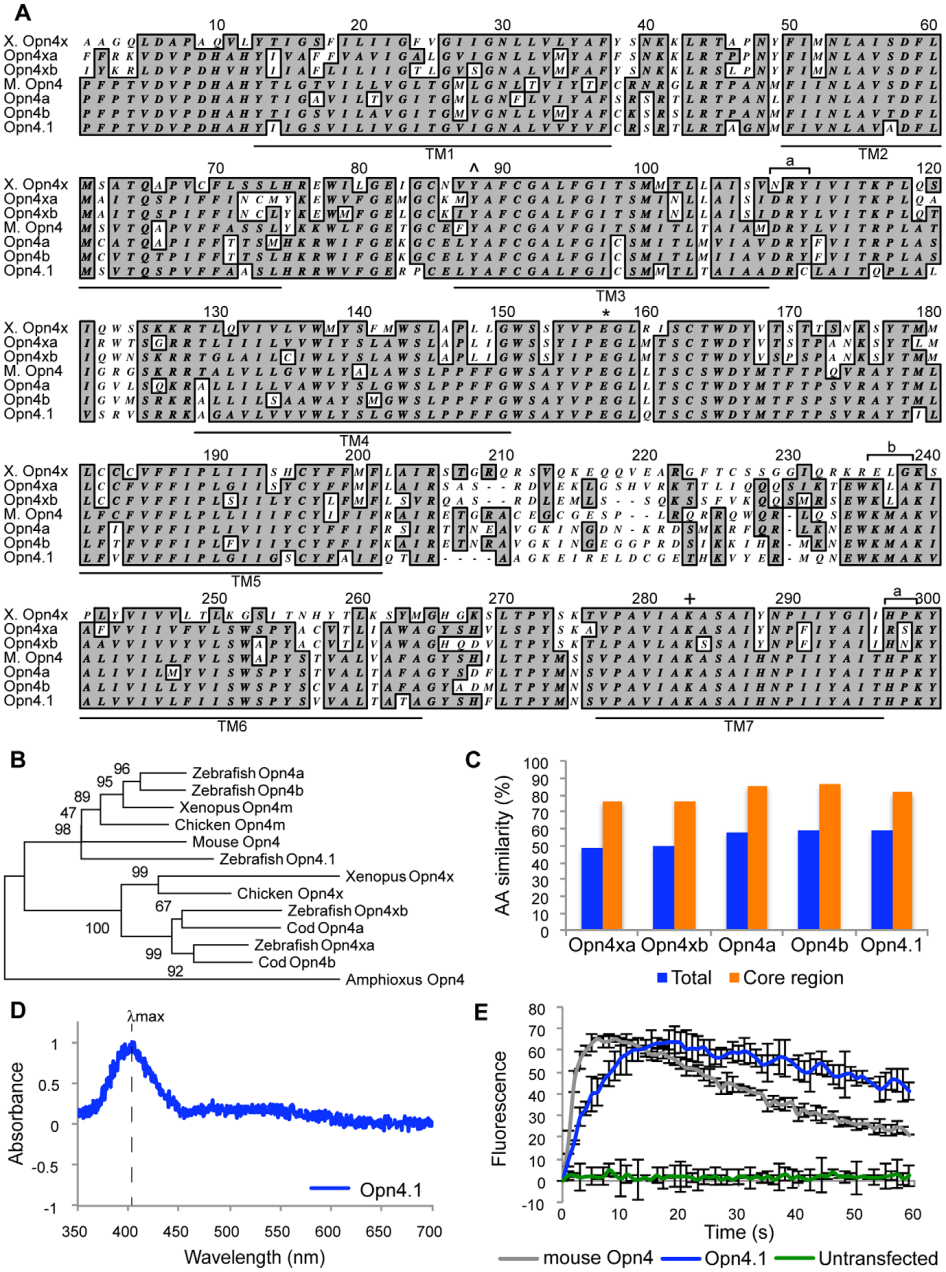
Zebrafish proteins share features of mammalian melanopsin. (A) Alignment of the core region of the predicted zebrafish melanopsin-related proteins with the Xenopus Opn4x and mouse Opn4 proteins. The core region includes seven transmembrane domains (TM1-7, underlined) and associated intracellular and extracellular loops. The DRY tripeptide motif of G-protein coupled receptors and the two signature motifs of rhabdomeric opsins are indicated by brackets (a, b and c, respectively). Glu (*) or Tyr (∧) is a possible counter ion for Schiff base linkage and Lys (+) is the site of chromophore binding. (B) Phylogenetic analysis separates the five zebrafish proteins into the Opn4m and Opn4x groups as indicated in a neighbor-joining tree rooted to Amphioxus Opn4 with five hundred bootstrap values. (C) Percentage of amino acid similarity between zebrafish Opn4-related and mouse Opn4 proteins (blue) or core regions (orange). (D) Normalized absorbance difference spectrum for zebrafish Opn4.1 with a maximum of 403 nm (dashed line). (E) Time course in seconds (s) of the response of HEK-293 cells (green) or transiently transfected with zebrafish Opn4.1 (blue) or mouse Opn4 (grey), as measured by fluorescent calcium imaging.

The mouse *melanopsin* coding sequence was used as a query against the zebrafish genome (Ensembl Zv6) and five distinct sequences with considerable homology were identified. Four genes were already localized to different chromosomes and we mapped the fifth gene to chromosome 10 using the LN54 radiation hybrid-mapping panel (data not shown) [25]. Phylogenetic analyses cluster the *melanopsin*-related genes of vertebrates into two separate groups, *opn4x* (Xenopus-related) and *opn4* (mammalian-related) [8]. Two of the zebrafish genes, *opn4a* and *opn4.1* had been previously identified as belonging to the mammalian-like *opn4* group [8], [24]. The nomenclature for the zebrafish genes has been revised based on their syntenic relationships (Figure 1), degree of similarity with the frog and mouse protein sequences (Figures 2A and B) and zebrafish nomenclature conventions (refer to Materials and Methods). Two *opn4x*-related genes (*opn4xa, opn4xb) and* two *opn4*-related genes (*opn4a* and *opn4b*) map to syntenic chromosomal regions. We speculate that the fifth gene, *opn4.1*, arose by retrotransposition since the entire *melanopsin* open reading frame is encoded by a single exon and there is no evidence of synteny with any of the other zebrafish genes or with the Xenopus, chicken and mouse *opn4* loci. As shown below, the *opn4.1* retrogene is expressed in the retina and likely encodes a functional photopigment.

The predicted proteins encoded by the five zebrafish genes show 48–60% similarity at the amino acid level to mouse Opn4 (Figures 2A and C). However, similarity within the core region (i.e., the seven transmembrane domains and their associated intracellular and extracellular loops) is much higher, ranging from 77–85% (Figure 2C). All five zebrafish proteins share the hallmarks of opsins; G-protein coupled receptors that bind chromophore (Figure 2A). These properties include seven helical transmembrane domains, a lysine on the seventh transmembrane domain for chromophore binding, conserved residues that could serve as the counter ion for chromophore binding via a Schiff base linkage, a structural DRY tripeptide motif in the third transmembrane domain and rhabdomeric opsin signature motifs on the third and fourth cytoplasmic loops (KMAK, HPKY, respectively) [1], [26]. Both Opn4x proteins contain amino acid substitutions in the cytoplasmic loops where the G-protein binds, suggesting that this subgroup may have altered binding affinity or activate different G-proteins. Another exception to a largely conserved overall structure is an amino acid substitution in the DRY motif of Opn4.1, changing it to DRC. A comparable tyrosine substitution to cysteine has been observed in other G-protein coupled receptors and is thought to have minimal effect on function [27], [28].

Despite its unique genomic origin, the *opn4.1* locus encodes an Opsin protein that is functional in a heterologous cell culture system and has an absorbance spectrum similar to mouse melanopsin (Figure 2D). Following transfection and expression in HEK293 cells (Figure 2E), Opn4.1 mediates light-dependent induction of calcium release. The kinetics of the calcium response are slower than those for mouse melanopsin and there is also a delay in deactivation of this zebrafish protein.

### Multiple neuronal cell types express *melanopsin* in the developing zebrafish retina

**Figure 3:**
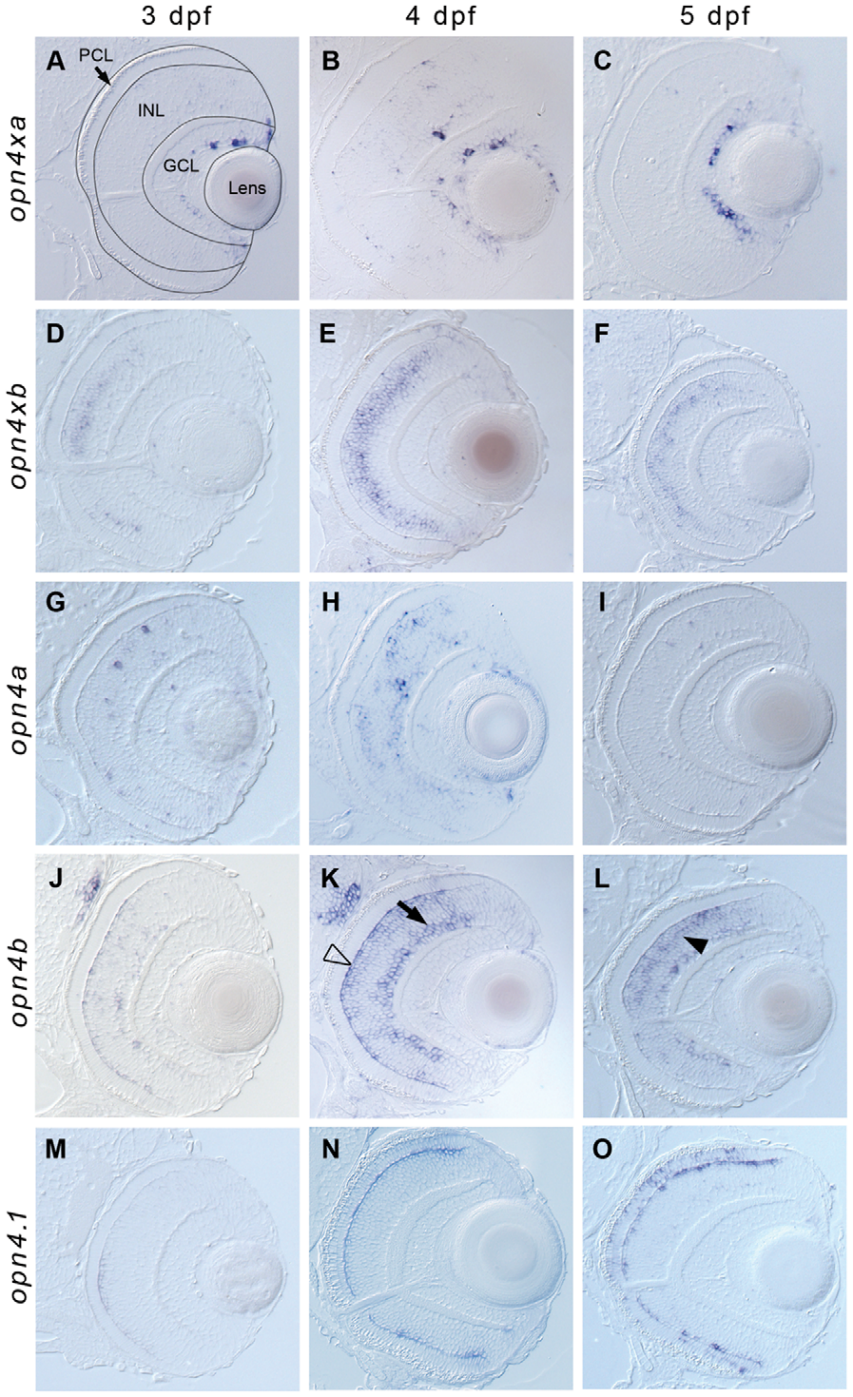
Diverse expression of zebrafish *opn4*-related genes in the developing retina. Profile of *opn4*-related gene expression in the larval retina from 3 to 5 dpf. Following whole-mount RNA in situ hybridization, larvae were embedded in LR gold media and 4 µm sections prepared. In A, the lens and retinal cell layers are indicated (GCL, ganglion cell layer, INL, inner nuclear layer, PCL, photoreceptor cell layer). (A–C) *opn4xa* is expressed in a small subset of cells in the ganglion cell layer. (D–F) *opn4xb* is transcribed in bipolar cells in a broad domain of the INL. (G–I) *opn4a* is expressed in clusters of bipolar cells scattered throughout the INL. (J–L) *opn4b* transcripts are found in three domains within the INL, where amacrine (arrowhead in L), bipolar (arrow in K) and horizontal cells (open arrowhead in K) are located. (M–O) *opn4.1* expression is weakly detected at 3 dpf but, one day later, strong expression is observed in horizontal cells in the outer shell of the INL. Sparse *opn4.1* expression is also found in the photoreceptor cell layer.

As in other vertebrates, the zebrafish retina is a multilayered structure composed of highly specialized neuronal cell types (Figures 3A and S1A). By 5 days post-fertilization (dpf), the larval retina has differentiated and is functional [21]. The zebrafish *opn4*-related genes are all expressed in the larval retina, but in distinct patterns. Only one Xenopus-related gene, *opn4xa*, shows expression similar to the mouse and human *opn4* genes, in a small subset of cells in the retinal ganglion cell layer [2], [3] (Figures 3A–C and S1B). These *opn4xa* transcripts colocalize with the ganglion cell marker, *gc56* [29] (Figure S1C).

Expression of *opn4xb* and all genes in the *opn4* group was detected in different regions within the inner nuclear layer (INL) (Figures 3D–O and S1D–J). The zebrafish *opn4xb* gene becomes uniformly expressed in the central region of the INL (Figures 3D–F) where bipolar cell bodies are located. Expression of *opn4a* was found in clusters of cells distributed throughout the INL (Figures 3G–I) although the pattern of expressing-cells varied for embryos reared under different light conditions (compare Figure 3I and Figure S1E and see below). In some cells in the INL of the retina, *opn4xb*, *opn4a* and *opn4b* were co-expressed with *bipolin*, a marker of bipolar cell identity [29] (Figures S1G–I). Strong expression of *opn4b* was also found in distinct sublaminae of the INL that correspond to regions enriched for amacrine or horizontal cells (Figures 3J–L). Transcripts for *opn4.1* localized to the outermost shell of the INL where horizontal cells are located and in scattered photoreceptor cells (Figures 3N and O).

### Expression of *melanopsin* genes in extraocular tissues but not in the larval pineal gland

In contrast to mammals where expression is confined to the retina, in non-mammalian vertebrates, *melanopsin* is not only expressed in the retina but also in discrete regions of the brain [1], [9], [11], [30]. The *opn4xa*, *opn4a* and *opn4b* genes are all expressed in non-overlapping patterns in the embryonic brain prior to retinogenesis (Figures S2A, D and H), which starts between 28–32 hpf [21]. The *opn4a* gene is expressed continuously from 1 to 3 dpf in the presumptive preoptic area, as defined by coexpression of the *orthopedia homolog* (*otp*) gene (Figures S2D–G). At 1 dpf, *opn4b* is expressed in an undefined region of the ventral forebrain and, commencing at 3 dpf, in bilateral domains in the thalamic region just dorsal to the lateral forebrain bundles (Figures S2H and I). The *opn4.1* gene is not expressed in the brain until 3 dpf, when transcripts are located at the juncture between the caudal hindbrain and anterior spinal cord, in cells in the ventricular region (Figures S2J and K).

Only one of the Xenopus-related genes is expressed outside of the retina. At 1 dpf, a small number (2–5) of *opn4xa*-expressing cells are bilaterally positioned in the dorsal diencephalon, in the proximity of the pineal complex (Figure S2A). The pineal gland is a photosensitive organ in non-mammalian vertebrates [31] and, in chickens, is a site of *melanopsin* expression [9], [11]. However, in zebrafish, *opn4xa* was not co-expressed with a marker of the developing pineal complex (*orthodenticle homolog, otx5* [32]) (Figure S2B). The *opn4xa*-expressing cells are also not located within the dorsal habenular nuclei (Figure S2C). These few cells reside at the base of the pineal stalk just medial to the dorsal habenular nuclei and persist until at least 5 dpf.

In addition to the developing brain, we find that only one gene, *opn4b*, is expressed outside of the nervous system at 1 dpf, in cells within trunk and tail somites (Figures S2L and M).

### Photoperiod modulates retinal *opn4* expression

**Figure 4:**
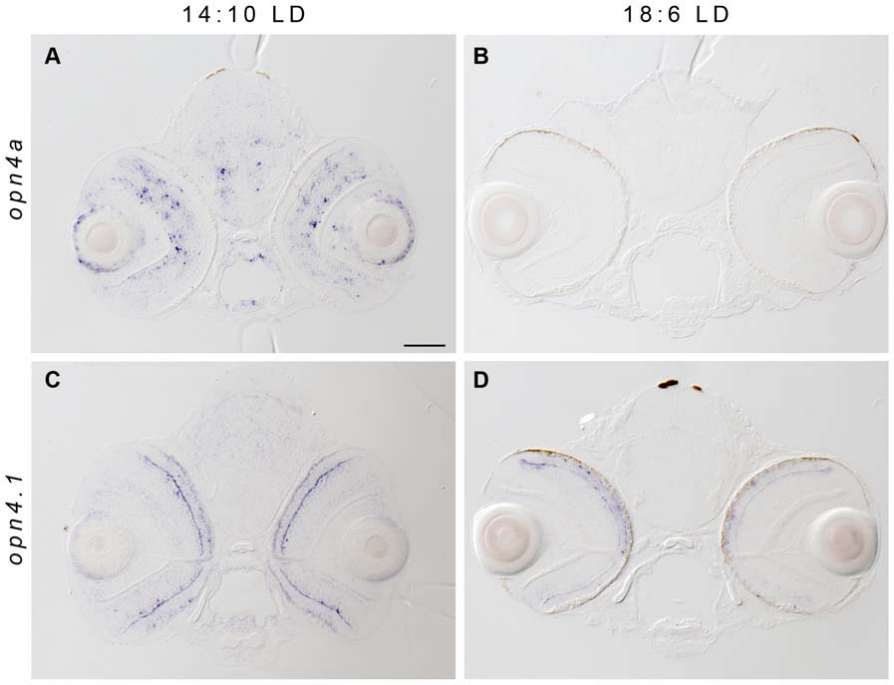
Photoperiod length influences *melanopsin* expression. (A, C) Larvae housed in 14∶10 LD or (B,D) 18∶6 LD cycles were fixed at ZT1 at 96 hpf, and assayed for *opn4*-related gene expression. *opn4a* and *opn4.1* expression in the inner nuclear layer is greatly reduced in the prolonged light conditions. Additionally, *opn4.1* transcripts are only detected in the photoreceptor cells (arrowhead) of larvae raised in the 14∶10 cycle. Over 90 larvae were assayed in 3 independent experiments.

Light adaptation in the retina is critical for signaling from rods and cones to ganglion cells across a wide range of light intensities. The expression of melanopsin in processing neurons of the inner nuclear layer of the zebrafish retina prompted us to examine the effect of different light lengths on transcription of *opn4*-related genes in vivo. We discovered that photoperiod length has a significant effect on expression of two *opn4*-related genes in the INL. Zebrafish larvae raised in two different light∶dark (LD) cycles, 14∶10 LD and 18∶6 LD, showed dramatic differences in expression of the *opn4a* and *opn4.1* genes at zeitgeber time 1 (ZT1) (Figure 4). Larvae kept in a 14∶10 LD cycle had transcripts in discrete regions of the retinal INL (Figures 4A and C). In addition, *opn4.1* expression was greatly increased in the photoreceptor cell layer (Figure 4C). However, *opn4a* and *opn4.1* transcripts were only weakly expressed in larvae maintained in an 18∶6 LD cycle, (Figures 4B and D).

**Figure 5:**
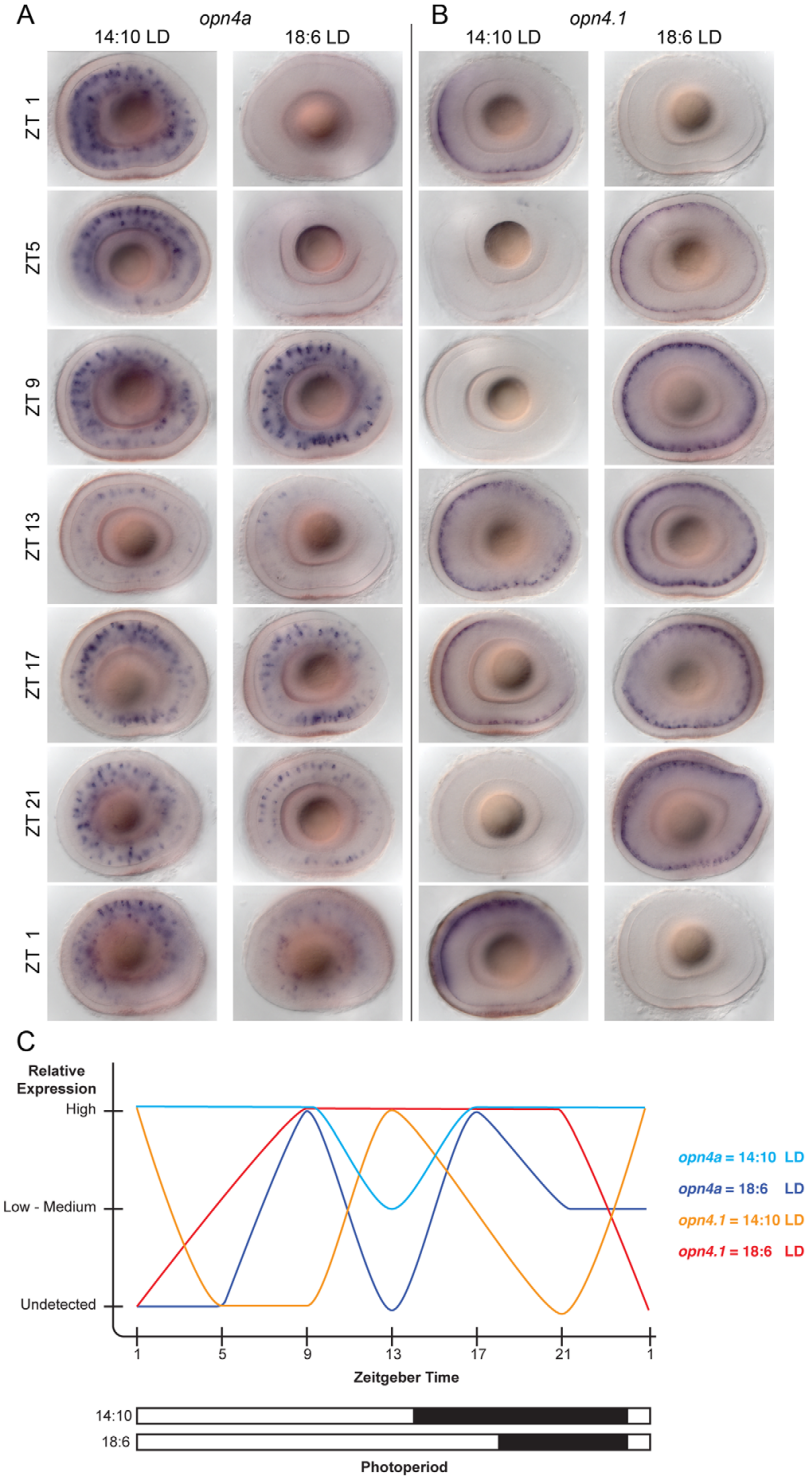
Rhythmic expression of *opn4a* and *opn4.1*. (A, B) Larvae maintained in 14∶10 LD or 18∶6 LD cycles were collected every 4 hours starting at ZT1. Expression of (A) *opn4a* and (B) *opn4.1* show diurnal rhythms with different phases for each light cycle. The rhythm of *opn4a* expression is a single wide waveform in 14∶10 LD, and either a dual waveform or an ultradian rhythm at 18∶6 LD. Under the same LD conditions, expression of *opn4.1* shows a reversal in the waveforms with respect to *opn4a*. (C) Summary graph representing the relative expression levels of *opn4a* and *opn4.1* under the two photoperiods depicted as white (light phase) and black (dark phase) bars.

To explore this finding further, we examined expression of the two genes at different phases during the 14∶10 and 18∶6 LD cycles and discovered that both show a robust diurnal rhythm that varies with each photoperiod and are not synchronous. The *opn4a* gene shows a decline in expression between ZT9 and ZT13 under both photoperiods, but expression decreases between ZT17 and ZT21 only in the 18∶10 LD cycle (Figure 5A). Thus, two peaks of expression are present under the prolonged light cycle (at ZT 9 and ZT17). Rhythmic expression levels of *opn4.1* also vary between the two photoperiods (Figure 5B). Under the shorter LD cycle, *opn4.1* shows two peaks of expression (ZT1 and ZT13), whereas a single peak of expression from ZT5 through ZT21 is found in the 18∶6 cycle. However, the phase of the diurnal rhythm is dramatically different between the expression profiles of *opn4a* and *opn4.1* in both photoperiods (Figure 5C). These results indicate that the melanopsin system in zebrafish is rhythmic and has the capability to respond to environmental light conditions by modulating gene expression.

## Discussion

Zebrafish larvae have evolved a sophisticated melanopsin system with distinct patterns of gene expression in multiple neuronal cell types in the retina, as well as in the brain and somites. As in mammals, perception of light by melanopsin may involve a subset of retinal ganglion cells in zebrafish, but additional expression in classical photoreceptors and in interneurons throughout the INL suggests that melanopsin has an expanded role in information processing in the developing retina of fish. Zebrafish retinal cells also have the capacity to regulate *opn4* expression based on the length of the photoperiod.

The literature on the *melanopsin* gene family of non-mammalian vertebrates has been complicated by the fact that the genes fall into two groups, those that encode proteins more similar to Xenopus melanopsin (Opn4x) or more similar to the human and mouse proteins (Opn4). It has been proposed that during evolution mammals lost the *opn4x* gene due to a chromosomal rearrangement [8]. The pairs of Xenopus-related (*opn4xa, opn4xb) and* mammalian-related (*opn4a* and *opn4b*) genes that exist in the zebrafish genome arose following the whole-genome duplication that occurred in the teleost lineage [33]. However, after the duplication event, considerable divergence in the regulatory sequences that control cell-type specific expression must have occurred because each pair of genes shows completely different patterns of expression. An additional unexpected finding, but one consistent with studies on the chicken [9] and Atlantic cod [10] is that only one gene from the Xenopus-related group, *opn4xa*, is transcribed in zebrafish retinal ganglion cells similar to the *opn4* gene in mammals. This suggests that a single ancestral *melanopsin* gene was expressed in the precursor cells to RGCs and that cell type-specific regulatory sequences were present at the *opn4* locus when the Xenopus-related gene was lost in mammals. Thus, the mouse *Opn4* and the zebrafish *opn4xa* loci may retain common cis-regulatory elements for transcriptional activation in the retinal ganglion cell layer. Whether the *melanopsin*-expressing RGCs of the zebrafish are functional equivalents of mouse ipRGCs, projecting to analogous regions of the brain and controlling circadian photoentrainment and sleep, remains to be demonstrated.

Because the fifth zebrafish gene, opn4.1, has a unique genomic structure and suspected origin as a retrogene, we wondered whether it encoded a functional protein. Not only is the gene expressed in the retina, but Opn4.1 protein produced and purified from a heterologous cell culture system also has properties of melanopsin. The absorption maximum of mouse melanopsin expressed in heterologous culture systems is shorter than that inferred from measurements of the action spectra of various light-dependent behaviors in mice that lack rod and cone photoreceptors [7], [34]. In this study, we found that zebrafish Opn4.1 has an even shorter λ_max_ than what has been reported for mouse melanopsin in cultured cell systems. It is likely that in its native form in vivo, the zebrafish protein has a longer λ_max_ than what is observed in vitro, but may still be blue shifted from the mouse protein. Other types of zebrafish opsins display a short-wavelength shift compared to homologous proteins from different species [35], [36] and it has been suggested that this shift correlates with optimization of the visual system for an aquatic habitat [37].

In addition to its absorbance spectrum, results from the calcium activation assay indicate that, when activated by light, Opn4.1 bound to the chromophore 11-cis retinal forms a visual pigment that functionally couples to a G-protein in HEK293 cells. There are some differences in the kinetics of this response compared to mouse melanopsin that probably reflect the variation between protein sequences. For example, the observed delay in deactivation of the zebrafish protein could be due to the reduced number of phosphorylation sites in the carboxyl tail of Opn4.1. These sites are thought to be important for deactivation of mouse melanopsin and are present in two of the other zebrafish Opn4-related proteins that show more similar kinetics to the mouse protein in the HEK293 assay system (Blasic and Robinson, unpublished observations). The results indicate that the protein encoded by *opn4.1* has the characteristics of a functional melanopsin photopigment. Its unique expression in classical photoreceptors suggests that the *opn4.1* retrogene coopted endogenous regulatory elements at its site of integration for transcriptional activation in the retina.

In contrast to mammals, where melanopsin is only expressed in the retina, non-mammalian vertebrates show expression in a variety of tissues [2], [9], [10], [11], [30] such as the skin, brain and pineal gland. It has been shown that zebrafish embryos are sensitive to light at 1 day, prior to retinogenesis [38], suggesting that production of photopigments in extraretinal tissues may contribute to early light detection. In post-natal mice, ipRGCs are the earliest photoresponsive cells [39], [40] and melanopsin is required for negative phototaxis at a stage when classical photoreceptors are not yet functional [41]. The products of *opn4a*, *opn4b* and *opnxa*, or other *opsin* genes that are known to be expressed in extraretinal tissues [42], [43], [44], could provide necessary light information to zebrafish embryos before the retina is completely functional.

Melanopsin was found in the pineal gland of chickens [9], [30], which is surprising because, structurally, pinealocytes are thought to be more similar to the ciliary photoreceptors of the vertebrate retina [14]. We did not detect *opn4*-related gene expression in the zebrafish pineal, at a stage when this organ is presumed to be light sensitive [45], [46]. However, a small number of *opn4xa*-expressing cells were found at another site in the epithalamus, at the base of the pineal stalk just medial to the bilaterally paired habenular nuclei. The habenula itself is known to express an *opn4x*-related gene in the Atlantic cod [9] and in the mouse is directly innervated by ipRGCs [47]. The close proximity of cells that express the RGC-specific *melanopsin* gene, *opn4xa*, to the presumptive pineal organ and habenular nuclei raises the intriguing possibility that they are specialized photosensitive projection neurons that interact with the adjacent structures.

In the zebrafish retina, expression of melanopsin-related genes encompasses all layers and neuronal types. In agreement with studies of other non-mammalian vertebrates [1], [9], [10], [11], two genes, *opn4b* and *opn4.1*, are expressed in the outer lamina of the INL in the horizontal cell layer. These results are also consistent with the observation that some horizontal cells are intrinsically photosensitive in fish [13], [48]. What was unexpected was the presence of *opn4a* and *opn4b* transcripts in bipolar cells because of their predicted evolutionary origin. It has been proposed that photoreceptive cells evolved from two parallel lineages in the retina, with rods, cones and bipolar cells derived from a ciliary photoreceptor precursor and amacrine, horizontal and retinal ganglion cells derived from a rhabdomeric photoreceptor precursor [49]. However, the finding of *melanopsin* expression in all of these cell types in the developing zebrafish retina argues that they arose from a common, evolutionarily ancient, bimodal ciliary/rhabdomeric photoreceptive precursor cell.

We discovered that the length of the photoperiod alters *melanopsin* expression in the retina. Reduced transcriptional levels of *opn4.1* in horizontal cells in response to a prolonged photoperiod could lead to differences in the ability of the retina to detect contrast because horizontal cells mediate the center surround responses necessary for contrast detection. In photoreceptors, as in ganglion cells [50], activation of the melanopsin pathway via Opn4.1 could lead to calcium release from internal stores and through gated calcium channels, thereby modulating calcium levels following extended exposure to light.

The *opn4.1* and *opn4a* genes show rhythmic expression but with different waveforms. When *opn4.1* expression peaks at ZT13 in horizontal cells under a 14∶10 LD cycle, *opn4a* expression is reduced in bipolar cells, and at some points when *opn4a* expression is high (e.g., ZT5 and ZT21), *opn4.1* transcripts are not detected. Notably, under the 18∶16 LD cycle, an opposite relationship is observed (e.g., expression of *opn4a* is undetected at ZT13, whereas *opn4.1* is highly expressed). Thus, rhythmicity of gene expression is maintained but is altered under different light∶dark conditions. The existence of independent diurnal rhythms for the two genes is suggestive of distinct oscillators functioning in horizontal and bipolar cells. Future experiments to monitor expression under constant dark conditions will determine whether *the circadian clock also regulates opn4a and opn4.1*. It is known in mammals that both melanopsin signaling and circadian activity within the retina alter electrical responses to light [51], [52]. The zebrafish studies suggest that rhythmic control of melanopsin is a potential mechanism to explain how both processes could be coupled to modulate physiology of the inner retina.

The influence of photoperiod length on *melanopsin* expression in horizontal cells and classical photoreceptors, combined with expression in bipolar and amacrine cells, illustrates the expanded role melanopsin signaling could play in the regulation of retinal circuitry. In mammals, ipRGCs signal back to dopaminergic amacrine cells [53] and are known to modulate visual processing by classical photoreceptors [54]. However, in fish and other non-mammalian vertebrates, expression of melanopsin in multiple neuronal types has the potential to influence retinal function directly in both an ipRGC-dependent and/or independent manner.

## Materials and Methods

### Zebrafish

Adult zebrafish of the Oregon AB strain [55] were housed in a 14∶10 light∶dark (LD) cycle at 27°C. All techniques and the care of zebrafish were approved by the Carnegie Institution Animal Care and Use Committee (Protocol #122). Embryos were obtained from natural matings, sorted at the 2–4 cell stage and initially maintained in rooms with different L∶D cycles. For the photoperiod experiment, 2–4 cell stage sibling embryos were divided into two groups. The first group was raised in a 14∶10 LD cycle and the second group was raised in an 18∶6 LD cycle. For both groups, larvae were fixed in 4% paraformaldehyde (PFA) at 96 hours post-fertilization (hpf), at a Zeitgeber time (ZT) of 1. To examine diurnal rhythms, we followed the same protocol and sacrificed larvae every four hours starting at 96 hpf until 120 hpf.

### Genomic and phylogenetic analyses

DNA sequence corresponding to the mouse *melanopsin* gene was used as a query against the zebrafish genomic database (Emsembl Zv6). Two *melanopsin* sequences were identified as corresponding to previously described genes (*opn4a* and *opn4b*) [8], [24]. Three new sequences, one in the *opn4m* group and two in the *opn4x* group, were identified with Expect (E) values ranging from 0.30 to 8.6×10^−15^. Because of its syntenic relationship and percent amino acid identity with *opn4a*, and based on the guidelines for naming of zebrafish genes (https://wiki.zfin.org/display/general/ZFINZebrafishNomenclatureGuidelines), the newly identified *opn4* group member is referred to as *opn4b* and the less conserved gene (previously called *opm4m2* in [8]) is renamed *opn4.1*. The two members of the *opn4x* group are referred to as *opn4xa and opn4xb*. Multiple sequence alignments of the Opn4 protein sequences were created using the ClustalW software [56] . Phylogenetic analysis was conducted using MEGA 4 [57]. Phylogenetic trees were obtained from the multiple sequence alignment using the neighbor-joining method with five hundred bootstraps.

### Isolation of zebrafish *melanopsin*-related genes

Total RNA was extracted from larvae at 5 dpf using TRIzol reagent (Invitrogen) and cDNA was synthesized using the RETROscript kit (Ambion). To isolate a unique fragment from each predicted zebrafish *melanopsin*-related cDNA, forward and reverse primers (Supplemental Experimental Procedures) were designed using the Primer3 program (http://frodo.wi.mit.edu/primer3) for amplification by the polymerase chain reaction (PCR). Appropriately sized PCR products were subcloned into the pCR II-TOPO vector using the TOPO TA cloning kit. The resultant plasmids were linearized and sense and antisense digoxigenin or fluorescein-labeled RNA probes were transcribed in vitro (for details see Table S1).

### RNA in situ hybridization

Whole-mount RNA in situ hybridization was carried out as in [32] with over 100 larvae assayed for each probe in multiple experiments. Hybridized probes were detected using alkaline phosphatase-conjugated antibodies (Roche Applied Science) and were visualized using nitro blue tetrazolium chloride and bromo-4-chloro-3-indolyl phosphate (NBT/BCIP) (Roche Applied Science). For double labeling, reacted specimens were stained in iodonitrotetrazolium violet and bromo-4-chloro-3-indolyl phosphate (INT/BCIP) (Roche Applied Science) as described [32]. Following in situ hybridization, embryos or larvae were post-fixed overnight in 4% PFA at 4°C, dehydrated in a 35%, 50%, 75% ethanol series and embedded in LR gold media (London Resin). Sections (4 µm) were prepared on a Leica ultramicrotome.

### Absorbance spectrum analysis

Full-length cDNA for *opn4.1* was amplified using the following primers sequences: *opn4.1* 5′-NNNNNNCAATTGATGAGCCATCACTCTTCATG-3′ and 5′NNNNNNGCGGCCGCTTAGGCAGGCGCCACTTGGCTGGTCTCTGTGTTCCCTCCAAGCAAAGCCT-3′. Sequences corresponding to the peptide from bovine rhodopsin recognized by the monoclonal antibody 1D4 [58] were included in the reverse primer. The *opn4.1* PCR product was subcloned into the pMT3 vector [59] and used in Lipofectamine 2000 (Invitrogen) transfection of HEK293 cells. Transfected cells producing zebrafish Opn4.1 or mouse Opn4 were harvested and stored at −80°C. Cells were resuspended in PBS and incubated with 40 µM 11-*cis*-retinal in the dark. Proteins were solubilized from cell membranes as described [60]. The ID4 monoclonal antibody 1D4 was used to purify melanopsin by immunoaffinity chromatography [61]. Purified melanopsin was eluted in 0.1% dodecyl maltoside in phosphate buffered saline and analyzed using a Hitachi Model U-3300 dual path spectrophotometer.

### Kinetic measurement of melanopsin activity by fluorescent Ca^2+^-imaging

The procedure for measuring melanopsin activity was modified from [62]. Transfected HEK-293 cells were allowed to grow for 24 hours and then released from the plate with 0.25% trypsin (Invitrogen), counted, and replated for fluorescent Ca^2+^-imaging at a density of 10^5^ cell per well in a 96 well plate with a clear bottom and black walls (BD). One day after re-plating, cells were washed twice with Hank's Balanced Salt Solution (HBSS) containing 20 mM HEPES, pH 7.4, and incubated in HBSS/HEPES supplemented with 4 µM Fluo-4 (Molecular Probes), 0.02% pluronic acid (Invitrogen), and 50 µM 11-*cis* retinal. Fluorescent measurements were performed on a Tecan Infinite M200 microplate reader (Tecan Group Ltd.,) (EX 485 nm, EM 520 nm) at a sampling rate of 1 Hz for 60 seconds. Background fluorescence was subtracted to account for variations in transfection efficiency and dye loading.

## Supporting Information

Figure S1 **Expression of zebrafish** ***opn4*** **-related genes in unique patterns in multiple retinal cell types.** (A) Schematic diagram of the multilayered retina of the zebrafish larval eye with the photoreceptor cell layer (PCL), inner nuclear cell layer (INL), ganglion cell layer (GCL) and lens indicated. (B–J) Whole mount single or double RNA *in situ* hybridization at 5 dpf with the indicated probes. (B,C) *opn4xa* is expressed in a subset of cells in the GCL that coexpress *gc56*. (D–F) *opn4xb*, *opn4a* and *opn4b* are all expressed in subregions of the INL and some cells (G–I) coexpress *bipolin* (*bip*), a marker of bipolar cells. (J) *opn4.1* is transcribed in horizontal cells in the outer lamina of the INL.
(PDF)

Figure S2 **Expression of** ***opn4*** **-related genes in extraocular tissues prior to retinogenesis.** (A) *opn4xa* is weakly expressed in bilateral domains the dorsal diencephalon as early as 1 dpf. (B) At 2 dpf, the *opn4xa*-expressing cells (blue) are located in close proximity to the *orthodenticle homolog 5 (otx5*) expressing presumptive pineal gland (C) and medial to the dorsal habenular nuclei that express the *Ca^2+^-dependent activator protein* (*cadps2*) at 4 dpf [63]. (D–F) From 1–3 dpf, *opn4a* positive cells are found in the forebrain where (G) they coexpress the *orthopedia* (*otp*) gene, a marker of the preoptic area. (H) At 1dpf, *opn4b* expression is found in the ventral forebrain and (I) at 4dpf is expressed in the dorsal thalamus (J–K) *opn4.1* is expressed in small subset of cells in the caudal hindbrain at 4dpf. *opn4b* is the only gene that is also expressed in the body, as shown in (L) a whole-mount embryo and in (M) a section through the tail region at 1dpf.
(PDF)

Table S1 (XLS)
