# Supplementary figures and images for: Unexpected Diversity and Photoperiod Dependence of the Zebrafish Melanopsin System
Source: PLoS One. 2011 Sep 22;6(9):e25111. doi: 10.1371/journal.pone.0025111 (PMC3178608; doi:10.1371/journal.pone.0025111)

Supplemental Figure 1

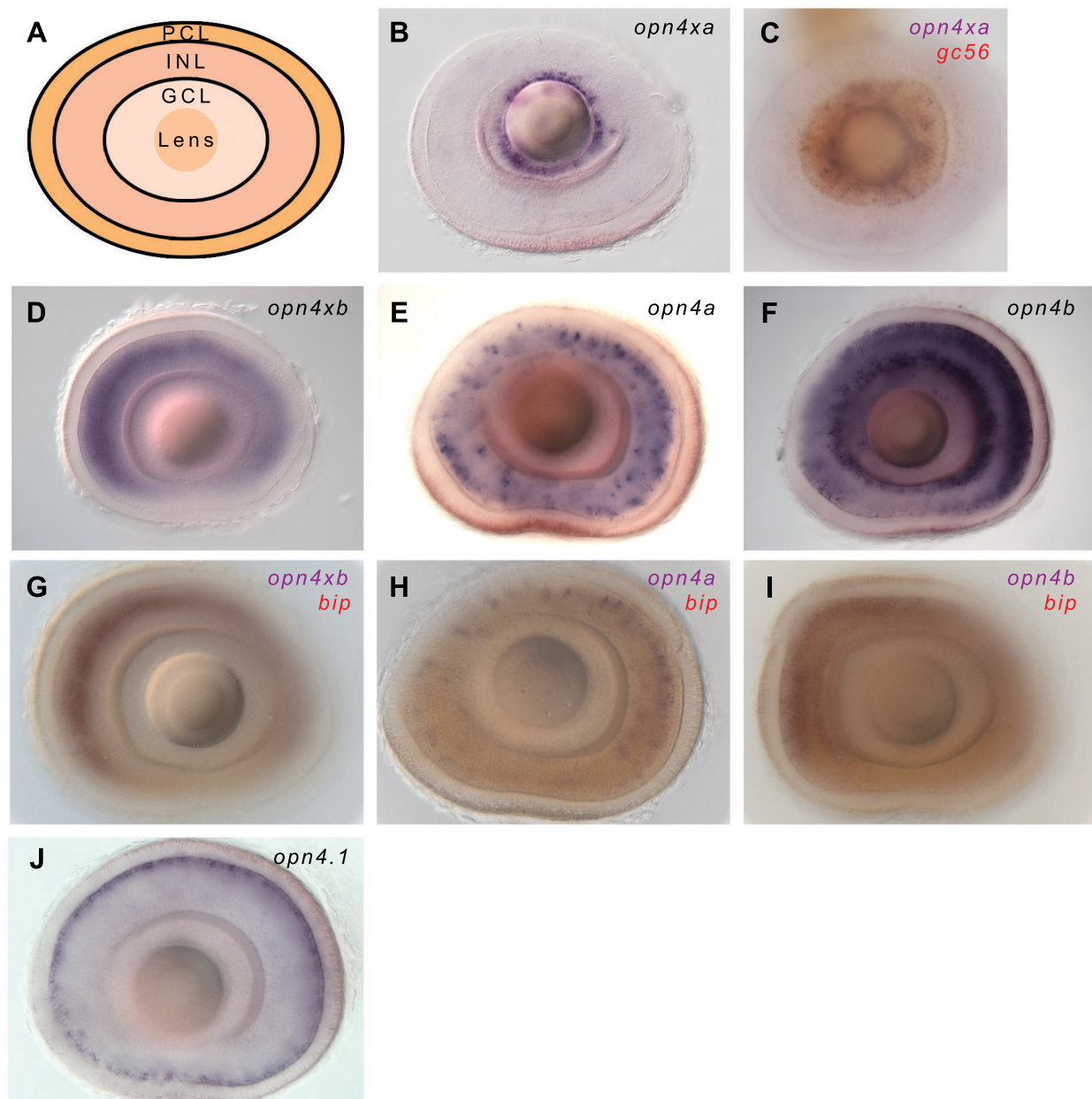

Supplement: Figure S1 — Expression of zebrafish opn4 -related genes in unique patterns in multiple retinal cell types. (A) Schematic diagram of the multilayered retina of the zebrafish larval eye with the photoreceptor cell layer (PCL), inner nuclear cell layer (INL), ganglion cell layer (GCL) and lens indicated. (B–J) Whole mount single or double RNA in situ hybridization at 5 dpf with the indicated probes. (B,C) opn4xa is expressed in a subset of cells in the GCL that coexpress gc56. (D–F) opn4xb, opn4a and opn4b are all expressed in subregions of the INL and some cells (G–I) coexpress bipolin (bip), a marker of bipolar cells. (J) opn4.1 is transcribed in horizontal cells in the outer lamina of the INL. (PDF) [file pone.0025111.s001.pdf]

Supplemental Figure 2

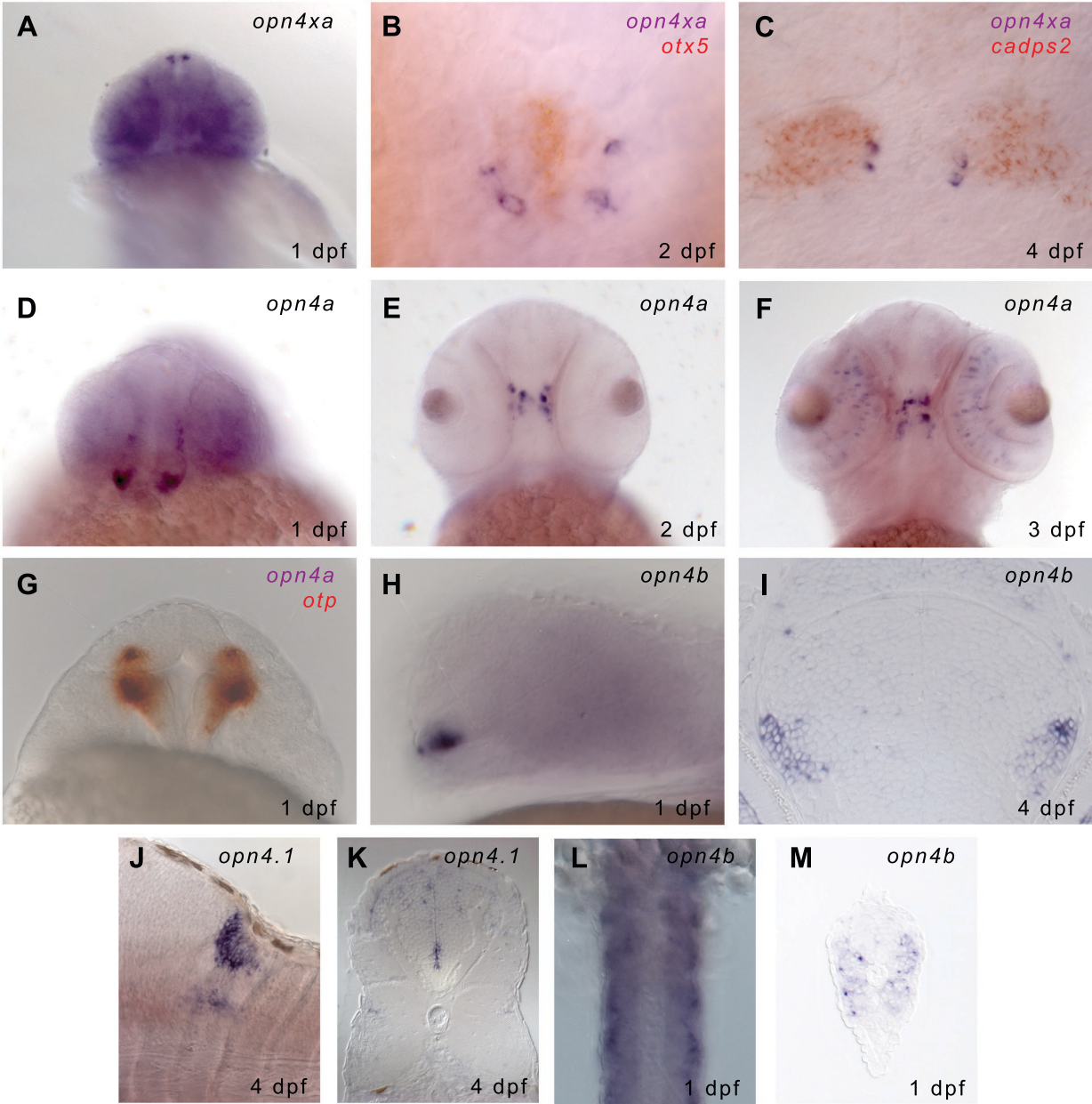

Supplement: Figure S2 — Expression of opn4 -related genes in extraocular tissues prior to retinogenesis. (A) opn4xa is weakly expressed in bilateral domains the dorsal diencephalon as early as 1 dpf. (B) At 2 dpf, the opn4xa-expressing cells (blue) are located in close proximity to the orthodenticle homolog 5 (otx5) expressing presumptive pineal gland (C) and medial to the dorsal habenular nuclei that express the Ca2+-dependent activator protein (cadps2) at 4 dpf [63]. (D–F) From 1–3 dpf, opn4a positive cells are found in the forebrain where (G) they coexpress the orthopedia (otp) gene, a marker of the preoptic area. (H) At 1dpf, opn4b expression is found in the ventral forebrain and (I) at 4dpf is expressed in the dorsal thalamus (J–K) opn4.1 is expressed in small subset of cells in the caudal hindbrain at 4dpf. opn4b is the only gene that is also expressed in the body, as shown in (L) a whole-mount embryo and in (M) a section through the tail region at 1dpf. (PDF) [file pone.0025111.s002.pdf]
